# mGem: Noncanonical nucleic acid structures—powerful but neglected antiviral targets

**DOI:** 10.1128/mbio.02730-25

**Published:** 2025-09-29

**Authors:** Václav Brázda, Richard P. Bowater, Petr Pečinka, Martin Bartas

**Affiliations:** 1 Institute of Biophysics of the Czech Academy of Sciences, Brno, Czech Republic; 2 School of Biological Sciences, University of East Anglia 6106, Norwich, United Kingdom; 3 Department of Biology and Ecology, Faculty of Science, University of Ostrava 206520, Ostrava, Czech Republic; Albert Einstein College of Medicine, Bronx, New York, USA

**Keywords:** DNA structure, G-quadruplex, Z-DNA, cruciform, targeting viruses

## Abstract

This perspective highlights the emerging significance of noncanonical nucleic acid structures—such as G-quadruplexes, Z-DNA/Z-RNA, and cruciforms—in viral genomes. Once considered structural oddities, these motifs are now recognized as critical regulators of viral replication, transcription, genome stability, and host–pathogen interactions. Despite mounting evidence of their functional relevance and therapeutic potential, these structures remain largely overlooked in virology and antiviral drug development. Their unique conformations offer highly specific molecular targets, with several small molecules already demonstrating the ability to modulate viral gene expression by stabilizing or destabilizing these motifs. The persistent underestimation of non-B DNA/RNA structures represents a missed opportunity in the fight against viral diseases. By synthesizing recent discoveries and emphasizing their biological and pharmacological promise, we aim to elevate awareness and catalyze interdisciplinary research. Harnessing the structural diversity of viral genomes could unlock novel antiviral strategies with high specificity and minimal off-target effects.

## NONCANONICAL NUCLEIC ACIDS IN VIRUSES

Viruses, as obligate intracellular parasites, rely on the host’s molecular machinery to replicate and propagate (1). While the canonical Watson–Crick double helix of DNA has long dominated our understanding of nucleic acid biology, it is now evident that viral genomes are massively diverse, as they can be formed by double-stranded molecules and frequently by single-stranded nucleic acids (Table 1). By “noncanonical nucleic acid structures” we refer to conformations that deviate from the classical right-handed B-DNA double helix or simple stem-loop motifs. Viral genomes can adopt a variety of noncanonical secondary structures in DNA, including G-quadruplexes (G4s), left-handed nucleic acids (Z-DNA), hairpins, and cruciforms. It has been demonstrated that these structures play critical roles in various essential functional and structural processes of viruses, including their genome organization, regulation of gene expression (2, 3), genome (in)stability (4), and host–pathogen interactions (5, 6). These structures, once considered biochemical curiosities, are now recognized as functionally relevant elements in both cellular and viral genomes (7). Despite this, their roles in viral life cycles remain underexplored, representing a significant gap in virology and antiviral research.

**TABLE 1:** Estimated percentage and example of viruses infecting bacteria, plants, and animals according to the Baltimore classification (8)^*a*^

Group (Baltimore classification)	Genome type and mRNA mechanism	Viruses infecting bacteria (bacteriophages)	Viruses infecting plants	Viruses infecting animals
I: dsDNA	Double-stranded DNA. mRNA is transcribed directly from DNA.	Dominant (typically 85–95%)—bacteriophage T4	Unknown (0%)	Very common (often 40–60%)—herpes simplex virus (HSV)
II: ssDNA	Single-stranded DNA (+/−). Genome is first converted to dsDNA.	Low percentage (a few %)—phage M13	Common (often 10–25%)—begomovirus	Less common (a few %)—parvovirus B19
III: dsRNA	Double-stranded RNA. mRNA is synthesized from dsRNA.	Very rare (almost 0%)—phage Φ6	Less common (a few %)—rice dwarf virus (RDV)	Less common (a few %)—rotavirus
IV: (+)ssRNA	Positive-sense single-stranded RNA. Genome serves directly as mRNA.	Very rare (almost 0%)—phage MS2	Dominant (often 60–70%)—tobacco mosaic virus (TMV), cucumber mosaic virus (CMV)	Very common (often 30–50%)—poliovirus
V: (–)ssRNA	Negative-sense single-stranded RNA. mRNA is synthesized from the (–) RNA template.	Unknown (likely 0%)	Common (often 10–20%)—tospovirus (tomato spotted wilt virus)	Common (often 15–25%)—influenza virus
VI: ssRNA-RT	Single-stranded RNA with reverse transcriptase. DNA is synthesized from RNA template.	Unknown (likely 0%)	Unknown (likely 0%)	Common (often 5–10%)—human immunodeficiency virus (HIV)
VII: dsDNA-RT	Double-stranded DNA with reverse transcriptase. DNA replicates via an RNA intermediate.	Unknown (likely 0%)	Extremely rare (almost 0%)—dahlia mosaic virus (DMV), cauliflower mosaic virus (CaMV)	Less common (a few %)—hepatitis B virus (HBV)

^*a*^ Percentages represent the approximate frequency of references in the literature (based on a curated PubMed search [9]) and are not an exhaustive coverage of natural diversity. The listed virions are representative examples where such structures have been identified or predicted.

In addition to structural motifs in DNA, RNA genomes and transcripts frequently fold into complex conformations that are similar to the noncanonical DNA structures. RNA G-quadruplexes (rG4s) are particularly relevant: in viruses, rG4s modulate essential processes such as genome replication, gene expression, and immune evasion (10). For example, rG4s in the HIV-1 genome affect reverse transcription and RNA packaging (11), while rG4s in the SARS-CoV-2 genome and host entry factors have been shown to influence viral entry and replication (12). Other noncanonical RNA elements, including hairpins, pseudoknots, and i-motifs, are increasingly recognized as dynamic regulators of viral life cycles.

**Fig 1:**
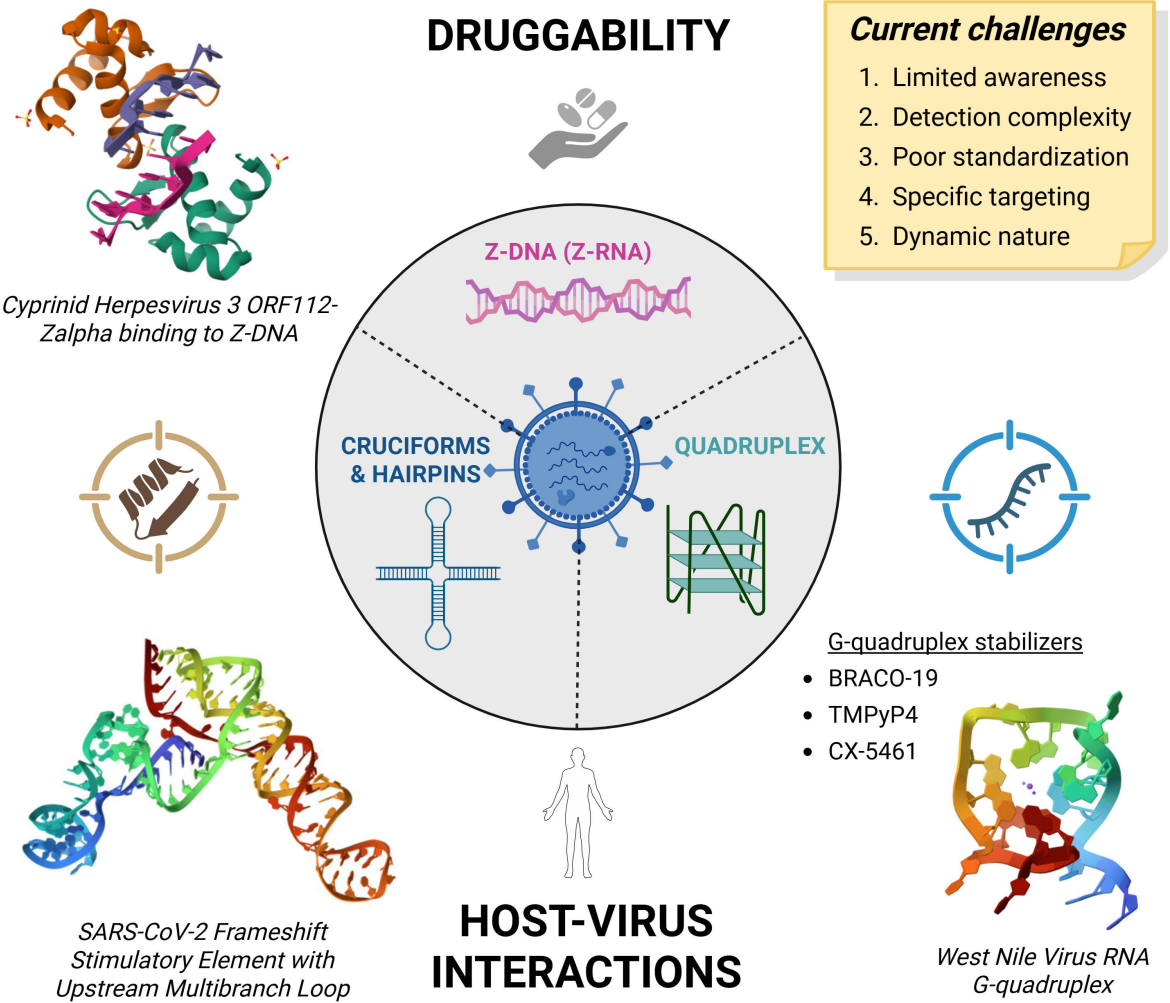
Noncanonical nucleic acid structures in viruses. A range of structures and functions has been proposed for sequences that are widespread in viral genomes, bringing therapeutic possibilities that are yet to be explored in detail. The following PDB structures are depicted: 4WCG (29), 8VCI (30), and 8UTG (31). Created with BioRender.com.

Recent advances in bioinformatics, structural biology, and chemical biology have enabled the identification and characterization of these noncanonical structures across a wide range of viral genomes (Fig. 1). These discoveries have revealed that such structures are not only present but also often conserved (13) and are functionally significant (14), influencing processes such as viral replication, transcription, translation, and immune evasion. For example, G4s have been identified in the genomes of HIV-1 (15), SARS-CoV-2 (16), Epstein-Barr virus (17), Rous sarcoma virus (18), hepatitis B (19, 20) and hepatitis delta virus (21), retroviruses (22), and herpesviruses (23), and they have also been shown to modulate transcriptional activity, genome packaging, and to play vital roles in viral-host coevolution (20, 24, 25). Despite their presence in various functional regions, noncanonical nucleic acid structures in viruses remain largely neglected as therapeutic targets. This oversight is particularly striking given the growing body of evidence supporting their functional importance and druggability (26). Small molecules that selectively bind and stabilize or destabilize these structures have shown promise in modulating viral gene expression and replication (27, 28). Moreover, the structural uniqueness of these motifs offers a level of specificity that is often difficult to achieve with traditional antiviral strategies.

## G-QUADRUPLEXES

G-quadruplexes, often abbreviated as G4s, are higher-order structures formed by guanine-rich sequences that stack into planar tetrads stabilized by Hoogsteen hydrogen bonding and monovalent cations (32). Recently, the G4 (Fig. 1) has been the most extensively studied noncanonical structure in viral genomes. G4s have been implicated in transcriptional regulation (33), genome replication (34), and recombination (35). In viruses, G4s have been shown to regulate the expression of key genes, including those involved in latency and immune evasion. For instance, in HIV-1, G4s in the long terminal repeat region modulate promoter activity, influencing viral latency and reactivation (36). Similarly, in herpesviruses, G4s are enriched in regulatory regions and may serve as epigenetic switches (37). More interestingly, it has been demonstrated that viruses that promote latent infections have a similar G4 propensity as the genome of their host (24, 25). By contrast, viruses that promote acute infections usually have G4-poor genomes (24) but are abundant in inverted repeats that can form hairpin and cruciform structures. Several experimentally solved G4 structures are available, including rG4 from West Nile virus genome (31). Knowledge about the exact structural shape of these nucleic acids can greatly facilitate the development of low-molecular-weight compounds that target them.

## Z-DNA/Z-RNA

Another intriguing structure is Z-DNA/Z-RNA, a left-handed helical form of nucleic acids that can arise under physiological supercoiling or high salt conditions (38). Z-DNA/Z-RNA has been implicated in innate immune sensing, particularly through interactions with Z-DNA binding protein 1 (ZBP1), which can trigger necroptosis in response to viral infection (39). However, coronaviruses have evolved efficient ways to evade ZBP1 sensing via utilizing their nsp15 protein, containing an endoribonuclease domain that cleaves viral RNA before it can be sensed by host ZBP1 (40). Studies have shown that some viruses, including *Poxviridae* and *Asfarviridae* families, encode proteins that bind Z-DNA/Z-RNA to evade immune detection (41). Recently, ZBPs were also identified to be encoded in the genomes of several giant viruses (42), and another recent study suggests that ZBP1 forms condensates with liquid–liquid phase separation properties upon viral infection (43). These findings suggest that Z-conformations are not only biologically relevant but are also actively targeted by viral countermeasures, underscoring their importance in host–pathogen dynamics.

## CRUCIFORMS AND HAIRPINS

Cruciform structures (and hairpins in the case of single-stranded genomes) can form within inverted repeat sequences, and these are another class of noncanonical motifs with potential relevance in virology. Cruciforms have been implicated in genome packaging, recombination, and transcriptional regulation in both prokaryotic and eukaryotic systems (44–46). In viruses, cruciforms may contribute to genome circularization, replication origin activity, or structural transitions during infection cycles, although direct evidence for any role that impacts viral life cycles remains limited (47). It was also recently found that sites of inverted repeats are a natural source of hot spot mutations in SARS-CoV-2 (48) and monkeypox viruses (49). In addition, parvoviruses and adeno-associated viruses use terminal hairpins as essential replication origins (50, 51), underscoring the biological significance of these noncanonical DNA structures in viral life cycles.

## METHODOLOGICAL APPROACHES

The identification of potential noncanonical structures in viral genomes has been accelerated by both computational prediction tools and experimental approaches. Algorithms such as G4Hunter (52), pqsfinder (53), G4RNA screener (54), and deep learning approaches such as DeepZ (55) allow for large-scale prediction of sequence motifs capable of forming higher-order structures (56). Experimentally, circular dichroism spectroscopy, nuclear magnetic resonance, and crystallography provide structural validation. High-throughput techniques such as SHAPE-MaP (57) and G4-seq enable transcriptome-wide mapping of RNA secondary and tertiary structures, including rG4s (58). Together, these complementary methodologies expand the landscape of accessible structural motifs in viral genomes.

## CONCLUSIONS AND CHALLENGES

The therapeutic potential of targeting noncanonical structures is increasingly supported by the development of structure-specific ligands. Several ligands, such as TMPyP4 (59), BRACO-19 (60), CX-5461 (61), QN-302 (62), and metallohelices (63), have been demonstrated to be able to bind (r)G4s and modulate gene expression in cancer and viral models (64). Importantly, some ligands exhibit selectivity for viral over host G4s, offering a promising avenue for antiviral drug development with a low level of off-target effects. Clinically approved compounds such as Topotecan and Berbamine have recently been shown to stabilize rG4s in genes encoding host entry factors and block SARS-CoV-2 pseudovirus entry *in vitro* and *in vivo* (12). Given the space constraints of this minireview, we cannot illustrate chemical structures of ligands here, but representative structures of widely used G4 ligands are available in comprehensive reviews (64, 65). Targeting Z-DNA/RNA is less advanced but equally promising: recent studies indicate that Korean red ginseng promotes cell death mediated by ZBP1, which helps to reduce the expression of viral proteins, thereby enhancing the host’s defense against the influenza A virus (66). Moreover, the integration of computational prediction tools with high-throughput screening platforms has accelerated the discovery of novel ligands and binding motifs (64).

Currently, the analyses and potential exploitation of noncanonical structures in viral genomes face several challenges. First, the dynamic and context-dependent nature of noncanonical structures complicates their detection and functional validation. Second, the diversity of methodologies for structure prediction and (high-throughput) ligand screening hinders cross-study comparisons. Third, the limited awareness of these structures among virologists has slowed their integration into mainstream antiviral research. Addressing these challenges will require interdisciplinary collaboration, combining expertise in virology, structural biology, computational modeling, and medicinal chemistry.

Beyond well-studied examples such as HIV-1 and herpesviruses, the distribution and functional impact of noncanonical structures in plant viruses, bacteriophages, and giant viruses remain largely uncharted. Given their ecological and medical importance, systematic surveys across underexplored viral families could uncover new structural vulnerabilities.

Integrating cryo-EM, transcriptome-wide structure probing, interactome mapping, and machine learning-based predictions will be essential to build a comprehensive atlas of viral noncanonical motifs. While ligand development has so far focused on G4s, advances in small-molecule design, synthetic biology, and CRISPR-based programmable nucleic acid targeting suggest that Z-conformations, cruciforms, and hairpins may also become pharmacologically tractable. Importantly, the conservation of certain motifs across viral families points to the possibility of developing broad-spectrum antivirals that exploit structural commonalities rather than sequence similarity. Realizing this potential will require not only interdisciplinary collaboration but also the establishment of community-wide standards and databases to enable reproducibility and cross-viral comparisons.

Noncanonical nucleic acid structures represent a rich and largely untapped frontier in virology. Their functional relevance, structural uniqueness, and druggability make them compelling targets for therapeutic innovation. As the field moves toward more precision-based antiviral strategies, expanding the research and therapeutic focus to include noncanonical nucleic acids across diverse viral systems could provide a new conceptual and practical framework for precision antiviral strategies.
